# Young Adults’ Loneliness and Depression During the COVID-19 Pandemic: A Moderated Mediation Model

**DOI:** 10.3389/fpsyg.2022.842738

**Published:** 2022-06-09

**Authors:** Fangyan Lv, Meng Yu, Jie Li, Jingbin Tan, Zhanhang Ye, Mengqi Xiao, Yalin Zhu, Siyuan Guo, Yanping Liu, Dingguo Gao

**Affiliations:** ^1^Department of Psychology, and Guangdong Provincial Key Laboratory of Social Cognitive Neuroscience and Mental Health, Sun Yat-sen University, Guangzhou, China; ^2^School of Computer Science and Technology, Guangdong University of Technology, Guangzhou, China; ^3^School of Educational and Technology, Guangdong Polytechnic Normal University, Guangzhou, China; ^4^School of Cultural Tourism and Geography, Guangdong University of Finance and Economics, Guangzhou, China

**Keywords:** protective factors, cognitive reappraisal, resilience, loneliness, depression

## Abstract

Since the outbreak of the COVID-19 pandemic in December 2019, millions of people have been infected with the disease. The COVID-19 pandemic also produced severe mental health problems, such as loneliness and depression. The present study aimed to examine the mediating role of cognitive reappraisal and moderating role of resilience in the relationship between young adults’ loneliness and depression during the pandemic by adopting a cross-sectional research approach. In March 2020, 654 young adults (18–29 years old) were recruited to complete the measures for loneliness, depression, emotion regulation, and resilience. Results found that loneliness was positively and moderately associated with depression (*r* = 0.531, *p* < 0.001), and that both loneliness and depression were separately negatively associated with cognitive reappraisal (*r* = −0.348, *p* < 0.001; *r* = −0.424, *p* < 0.001) and resilience (*r* = −0.436, *p* < 0.001; *r* = −0.419, *p* < 0.001). The results indicated that both loneliness and depression were not associated with expressive suppression (*r* = 0.067, *p* = 0.087; *r* = −0.002, *p* = 0.961). The moderated mediation model results revealed that only cognitive reappraisal partially mediated the relationship between loneliness and depression (*b* = −0.301; *Boot* 95% *CI* = −0.388, −0.215). In addition, the results of the moderated mediation model indicated that resilience moderated the association between loneliness and depression (*b* = 0.035, *p* < 0.001, *Boot 95% CI* = 0.014, 0.055), while also moderated the impact of cognitive reappraisal on depression (*b* = −0.031, *p* < 0.001, *Boot 95% CI* = −0.058, −0.005). These findings have practical implications that broaden our understanding of depression in young adults and shed light on how to enhance cognitive reappraisal and resilience as a means of combating depression in this age group during the COVID-19 pandemic.

## Introduction

The COVID-19 pandemic has been declared as a public health emergency (WHO, 2020). The disease not only heightened the risk of death, but also caused mental health problems in China and the rest of the world (Bao et al., 2020; Cao et al., 2020; Duan and Zhu, 2020; Xiang et al., 2020). Since the virus began to spread in early 2020, the threat of death from infection, strict social distancing regulations, and the delayed opening of schools and universities across China have had an inevitable negative impact on mental health and led to an increase in loneliness, death anxiety, and depression among the general public (Chen et al., 2020; Hoffart et al., 2020; Li and Wang, 2020; Liu et al., 2020; Qiu et al., 2020; Wickens et al., 2021; Wu et al., 2021a), medical staff (Wang J. et al., 2020), college students (Cao et al., 2020; Fu et al., 2021; Ramo and Lim, 2021), and the older (Ogrin et al., 2021). During the pandemic, loneliness has been a common occurrence among those who are socially isolated (Pietrabissa and Simpson, 2020; Smith and Lim, 2020; Tull et al., 2020; Mansour et al., 2021; Wickens et al., 2021), especially young adults (Groarke et al., 2020; Ramo and Lim, 2021). Although transient loneliness does not lead to psychological and behavioral disorders, long-term or severe loneliness may result in certain emotional disorders and deteriorating mental health (Wang et al., 2018). Loneliness was thus connected to a series of negative physical and mental health problems (Holt-Lunstad et al., 2015; Lim et al., 2016; Palgi et al., 2020). Previous study has shown that loneliness at an earlier time point could predict depression and social anxiety at subsequent time points (Lim et al., 2016). However, in the earlier time of the COVID-19 pandemic when death threats and negative emotions were diffused, individuals were more anxious of infecting the COVID-19 virus rather than social anxiety. In addition, studies have indicated that among the factors associated with loneliness, depression has the greatest impact (Fuente et al., 2018).

Although scholars have not yet agreed on whether there is a causal relationship between loneliness and depression, the current study adopted the hypothesis that loneliness is a risk predictor of depression. Some studies have shown that loneliness and depression may be mutually (Cacioppo et al., 2006), but others have argued that loneliness is a notable result of depression (Erzen and Cikrikci, 2018). Longitudinal research has also demonstrated that loneliness predicts depression (Chang, 2017), not only at a specific moment but also vertically in time (Louise et al., 2010; Qualter et al., 2010; Vanhalst et al., 2012). Moreover, studies have revealed an association between loneliness and depressive symptoms (Cacioppo et al., 2006; Chang, 2017) and shown that loneliness can predict a heightening of depressive symptoms over time (Fuente et al., 2018). Recently, studies have revealed that loneliness has a significant effect on depression at a moderate level (Erzen and Cikrikci, 2018). In addition, many researchers have become interested in exploring the relationship between loneliness and depression among young adults (Cacioppo et al., 2006; Richardson et al., 2017; Groarke et al., 2021). In younger individuals, loneliness is tied to abnormal coping strategies adopted to deal with emotional problems (Vanhalst et al., 2012), which makes this group more prone to depression (Van Winkel et al., 2017; Kuczynski et al., 2021). For example, ruminant thinking (Zhang et al., 2019) and coping strategies (Fuente et al., 2018) played mediating roles in the relationship between loneliness and depression. The link between loneliness and depression has been made especially clear throughout the COVID-19 pandemic (Elmer et al., 2020; Hoffart et al., 2020; Misirlis et al., 2020; Wu et al., 2021b). Evidence has confirmed that young adults are vulnerable to loneliness and indicated an increased level of loneliness among young adults due to the pandemic (Lisitsa et al., 2020; Padmanabhanunni and Pretorius, 2021). In these cases, induced loneliness caused by the pandemic was significantly connected with depression (Elmer et al., 2020; Rossi et al., 2020). Thus, loneliness could be viewed as a predictor of depression (Rossi et al., 2020; Santini et al., 2020; Thakur and Jain, 2020).

Another aim of this study was to explore the roles that emotion regulation and resilience had played in the lives of young adults during the COVID-19 pandemic. The protective factors of emotion regulation could be considered as important psychological resources. Psychological resources can help mediate individual’s responses to traumatic experiences (Conversano et al., 2020; Giuseppe et al., 2020; Guicciardi and Pazzona, 2020; Rossi et al., 2020), and then individual’s potential adaptive defense mechanism could help them overcome traumatic experiences brought on by COVID-19. Adaptive emotion regulation strategies have been shown to protect individuals who are exposed to community-based disasters (Ehring and Quack, 2010). The process model of emotion regulation was developed by Gross and John (2003) and includes two types of emotion regulation strategies. One of the strategies is cognitive reappraisal, which is an antecedent-focused strategy that reduces negative emotional effects, consciously changes the interpretation of emotion-evoked events and focuses on positive aspects of the situation (Gross and John, 2003). The other emotional strategy is expressive suppression, which is a response-focused strategy that individuals tries to inhibit any external cues related to their emotion state when their internal emotional responses have already been produced (Gross and John, 2003). Although loneliness is a negative emotional experience, commonly used adaptive regulation strategies (i.e., cognitive reappraisal) are associated with improved well-being and lower levels of loneliness (Kearns and Creaven, 2017). Previous studies indicated that the inability to regulate daily emotion responses was shown to be significantly associated with mental and behavioral problems, lower levels of resilience (Webb et al., 2012), and the development of depression (Ehring and Quack, 2010). Emotion regulation difficulties have also been linked to greater levels of loneliness (Gonçalves et al., 2019; Visted et al., 2019; Groarke et al., 2021) and were treated as predictors of loneliness in the context of the COVID-19 pandemic (Groarke et al., 2021). Researches have also revealed that emotion regulation strategies played a mediating role in the relationship between interpersonal stress and depression among undergraduate students (Moriya and Takahashi, 2013). Generally, cognitive reappraisal, as a adaptive strategy, may be considered as a psychological resource for young adults affected by COVID-19 pandemic (Kuhlman et al., 2021), whereas expressive suppression, as a maladaptive strategy, was associated with negative outcomes such as more negative affect and depression (Tyra et al., 2021). Cognitive reappraisal has been found to be negatively related to depression (Joormann and Stanton, 2016; Picó-Pérez et al., 2017; Sachs-Ericsson et al., 2021). And researchers have identified cognitive reappraisal as an important protector that has helped prevent young adults who are impacted by COVID-19 or other instances of prolonged stress from developing mental health issues (i.e., depression, anxiety, and sleep problems) (Xu et al., 2020; Kuhlman et al., 2021). Considering the association between cognitive reappraisal and depression and the fact that loneliness is a risk factor of depression (Rossi et al., 2020; Santini et al., 2020; Thakur and Jain, 2020), therefore, it is theoretically possible that cognitive reappraisal could influence the association between loneliness and depression. Previous research has found that the emotion regulation strategies played a mediation role in college students’ depressive symptoms during the COVID-19 pandemic (Ye et al., 2022). However, another study found that cognitive reappraisal was negatively associated with anxiety and depression, but expressive suppression was not associated with them during the early COVID-19 pandemic (Tyra et al., 2021). Considering both emotion regulation strategies may be used to cope with the influences from COVID-19, mediation analyses would be conducted to explore the impact of both emotion regulation strategies (i.e., cognitive reappraisal and expressive suppression) on the relationship between loneliness and depressive symptom. Therefore, we hypothesized that emotion regulation played a mediating role in the relationship between loneliness and depression in young adults.

Resilience is an important psychological resource that could also influence the association between loneliness and depression in young adults. However, few studies have explored the role of resilience in this relationship. Resilience is defined as “the process of adapting well in the face of adversity, trauma, tragedy, threats or even significant sources of threat” (American Psychological Association [APA], 2020). Generally, resilience is a positive quality that allows individuals to face adversity and is considered to be a protective mechanism that individuals can use to maintain mental health in the face of stress and trauma (Michael, 1987; Wu et al., 2020). The risk-protective model suggests that the adverse impacts of risk factors on an individual’s health status can be mitigated by resilience (Garmezy et al., 1984). In addition, low level of resilience to stress has been shown to correlated with an increased lifetime risk of antidepressant and anxiolytic drug use (Hiyoshi et al., 2015; Ran et al., 2020). Resilience could thus be a crucial component of reducing stress and psychological pain during traumatic events. Empirical results have indicated that resilience is negatively associated with depressive symptoms (Liu et al., 2015; Ye et al., 2020) and plays a protective role by reducing the influence of stress and the negative effects of depression (Kukihara et al., 2014; Liu et al., 2015). Furthermore, resilience has been shown to play a mediating role in the relationship between loneliness and depression among older adults in nursing homes (Zhao et al., 2018). Throughout the COVID-19 pandemic, depressive symptomology has been negatively correlated with resilience (Ye et al., 2020). In addition, resilience not only moderated the association between chronic pain and depression (Bauer et al., 2016) but also acted as a potential moderator in cases where individuals struggle with loneliness and sleep problems related to the COVID-19 pandemic (Grossman et al., 2021). In other words, the role of resilience buffers the deterioration of depression in individuals. Therefore, resilience could be considered as one of the protective factors that could moderate the relationship between loneliness and depression in young adults during the COVID-19 pandemic.

**FIGURE 1:**
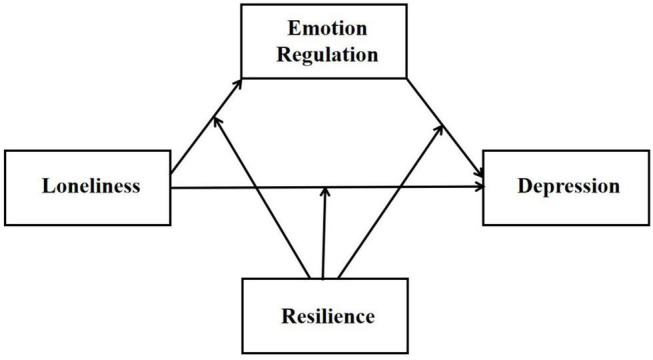
The proposed moderated mediation model.

In summary, this study aimed to construct a model to explore the impact of cognitive reappraisal and resilience on loneliness and depression in young adults during the COVID-19 pandemic. Thus, we hypothesized (1) higher levels of loneliness was positively associated with higher risk of depression symptoms, (2) emotion regulation strategies and resilience were negatively associated with loneliness and depression, (3) when not considering the buffering effect of resilience, loneliness predicted the risk of depressive symptoms through emotion regulation strategies (mediation), and (4) loneliness predicted the reduced risk of depression symptoms through both emotion regulation strategies (mediator) and resilience (moderator) (see Figure 1). In this study, no specific hypothesis was made regarding the mediating effect of emotion regulation strategies. The mediating role of suppression and reappraisal would be examined, seperately.

## Materials and Methods

### Participants

In March 2020, this study recruited randomly 654 participants online from three colleges located in Guangdong Province, China. The participants included 325 males (49.694%) and 329 females (50.306%) aged 18–29 (*M* = 19.980, *SD* = 1.801). Moreover, 98.471% of sample consisted of undergraduate students, and about three-quarters of the participants (72.02%) had a family income of more than $300 per head.

### Procedure

All of our data was collected using a web-based survey designed through an online survey platform called Wenjuanxing. Informed consent was collected at the beginning of the survey, and it was clear to participants that they could withdraw from the investigation at any time. Participants could work through the questionnaire at their own pace, and they could only move on to the next page once they had completed all of the items on the page they were currently working through. Before completing the final online survey, there was a pre-test. The questions included in the pre-test did not appear in the final survey in order to allow for the identification and correction of any possible errors in the questionnaire. This study was approved by the Institutional Review Board at first author’s affiliation.

### Measures

#### Demographic Information

The demographic variables were measured in this study which included gender, age, marital status (single, married, divorced, widowed), education level (undergraduate, upgraduate), region (urban, rural), and self-rating health [from 1 (“very bad”) to 5 (“very good”)], see Table 1.

**TABLE 1:** Demographic characteristics of the sample.

Characteristic		n (%)	UCLS-8 (*M* ± *SD*)	SDS (*M* ± *SD*)
Gender	Male	325 (49.694)	16.169 ± 4.541	35.068 ± 7.390
	Female	329 (50.306)	16.660 ± 4.531	35.055 ± 7.376
Age (years)	>20	474(72.477)	16.399 ± 4.630	35.156 ± 7.603
	21–29	180 (27.523)	16.461 ± 4.303	34.811 ± 6.759
Region	Urban	357 (54.587)	16.499 ± 4.485	34.950 ± 7.183
	Rural	297 (45.413)	16.317 ± 4.609	35.195 ± 7.614
Education	Undergraduate	644 (98.471)	16.443 ± 4.543	35.028 ± 7.372
	upgraduate	10 (1.529)	14.700 ± 4.165	37.200 ± 7.800
Marital Status	Married*^a^*	10 (1.529)	14.700 ± 4.165	37.200 ± 7.800
	Unmarried	644 (98.471)	16.443 ± 4.543	35.028 ± 7.372
Smoking	Yes	25 (3.823)	16.400 ± 4.752	39.840 ± 9.831
	No	629 (96.177)	16.417 ± 4.535	34.871 ± 7.208
Drinking	Yes	65 (9.939)	16.723 ± 4.665	38.785 ± 8.907
	No	589 (90.061)	16.382 ± 4.528	34.650 ± 7.078
Self-rated health	Bad or average	60 (9.174)	19.870 ± 4.073	43.283 ± 7.951
	Good	199 (30.428)	17.472 ± 4.163	35.749 ± 6.935
	Very good	395 (60.398)	15.360 ± 4.411	33.466 ± 6.594

*^a^Including married, divorced, and widowed.*

#### Depression

Depression was measured according to the Chinese version of the Self-Rating Depression Scale (SDS), which includes 20 items. Each item was rated on a 4-point scale (1 = *never*, 4 = *always*). Higher scores indicated a higher level of depression, and there was a good reliability that the Cronbach’s α coefficient for the SDS was 0.94.

#### Loneliness

Loneliness was measured according to the Chinese version of the short-form version of the University of California Los Angeles’ Loneliness Scale (UCLS-8), which consists of eight items. Each item was rated on a 4-point scale (1 = *never*, 4 = *always*). Higher scores indicated a higher level of loneliness, and the Cronbach’s α coefficient for the UCLS-8 was 0.87.

#### Resilience

To measure resilience, we used the Chinese version of the Connor-Davidson Resilience Scale (CD-RISC10), which includes 10 items. Each item was rated on a 5-point scale (0 = *never*, 4 = *always*), with higher scores indicating a higher level of resilience. The Cronbach’s α coefficient for the SDS was 0.88.

#### Emotion Regulation

Emotion regulation strategies were measured using the Chinese version of the Emotion Regulation Questionnaire (ERQ) developed by Gross and John (2003), which consists of 10 items that cover cognitive reappraisal and expressive suppression or which consists of 10 items, including cognitive reappraisal and expressive suppression. Each item was rated according to a 7-point scale ranging from 1 (*strongly disagree*) to 7 (*strongly agree*). Higher scores suggested a greater tendency to use a certain strategy. For the present sample, the Cronbach’s α coefficient was 0.71 for the expressive suppression scale and 0.76 for the cognitive reappraisal scale.

#### Analyses

In this study, there was not an extensive amount of missing value data in the participants’ responses, so no data were deleted. We used SPSS 23.0 for data analyses, and set the *p*-value threshold at 0.05 (two-tailed) for statistical significance. First, we calculated descriptive statistics and correlations for the interested variables. Secondly, we separately calculated the mediation effect and the moderated mediation effect using Hayes’s PROCESS windows (Model 4, Model 59) (Hayes, 2013) to further explore the relationship of the interest variables (Figure 1). The covariates included age and gender. The non-parametric bootstrap method was used to test mediation effects with 5000 resamples. And finally, the simple slope analysis was used to further explore the moderation effect.

## Results

### Descriptive Statistics

Table 1 indicated the demographic data collected from all participants. We found that self-rated health were associated with loneliness (*r* = 0.355, *p* < 0.001) and depression (*r* = 0.319, *p* < 0.001) which indicated individuals with worse physical health might have higher level of loneliness and depression in this study where the scores of depression just indicated a risk tendency to be depressed rather than a clinically significant level of depression.

The results were depicted in Table 2, including means, standard deviations, and correlations for all of the variables in the study. Our findings suggested that loneliness in young adults was positively correlated with depression (*r* = 0.531, *p* < 0.001), and that both loneliness and depression in this age group were negatively associated with cognitive reappraisal (*r* = −0.348, *p* < 0.001; *r* = −0.424, *p* < 0.001) and resilience (*r* = −0.436, *p* < 0.001; *r* = −0.419, *p* < 0.001). However, the results indicated that none of loneliness (*r* = 0.067, *p* = 0.087), depression (*r* = −0.002, *p* = 0.961) and resilience (*r* = 0.055, *p* = 0.156) in this study were associated with expressive suppression.Therefore, the young adults in this study who frequently used cognitive reappraisal rather than expressive suppression were more likely to have higher levels of resilience. Therefore, cognitive reappraisal and resilience were negatively associated with loneliness and risk of depression symptom. Hypotheses 1 and 2 were thus supported.

**TABLE 2:** Descriptive statistics and correlations between variables.

	M	*SD*	1	2	3	4	5	6	7
1. Loneliness	16.416	4.539	−						
2. Resilience	18.651	4.175	−0.436***	−					
3. CR	29.622	5.560	−0.348***	0.341***	−				
4. ES	15.520	4.300	0.067	0.055	0.165***	−			
5. Depression	35.061	7.377	0.531***	−0.419***	−0.424**	–0.002	−		
6. Age	19.980	1.801	–0.001	–0.003	0.052	0.021	–0.018	−	
7. Gender			0.054	−0.132**	–0.028	−0.191***	–0.001	–0.073	−

CR, cognitive reappraisal; ES, expressive suppression. **p < 0.01. ***p < 0.001.

### Testing for Mediation Effect

As shown in Tables 3, 4, mediation analysis was conducted using PROCESS windows (Model 4) in SPSS in order to examine whether emotion regulation strategies mediates the association between loneliness and depression. Table 3 revealed the mediating role of cognitive reappraisal. As predicted, loneliness was significantly associated with depression, *b* = 0.866, *p* < 0.001, 95% *CI* = [0.760, 0.972] (Model 1). The results show that loneliness significantly negatively predicts cognitive reappraisal, *b* = −0.426, *p* < 0.001, 95% *CI* = [−0.514, −0.338] (Model 2). As Model 3 demonstrates, cognitive reappraisal significantly negatively predicted loneliness, *b* = −0.364, *p* < 0.001, 95% *CI* = [−0.452, −0.276]. The direct effect of loneliness on depression was also significant (Model 3), *b* = 0.711, *p* < 0.001, 95% *CI* = [0.603, 0.819]. We generated 5000 bootstrapping samples from the original data set (n = 654). The indirect effect of loneliness on depression via cognitive reappraisal was significant, *b* = 0.155, 95% Boot *CI* = [0.102, 0.216], *p* < 0.001. The direct effect was also significant, *b* = 0.711, 95% *Boot CI* = [0.603, 0.819], *p* < 0.001. The mediation effect accounted for 17.90% of the total effect. However, the results did not show the mediating role of expressive suppression in Table 4. Although the results showed that loneliness was associated with expressive suppression, *b* = 0.073, *p* = 0.044, 95% *CI* = [0.002, 0.145] (Model 2) and the direct effect of loneliness on depression was also significant in Model 3, *b* = 0.871, *p* < 0.001, 95% *CI* = [0.765, 0.978], but expressive suppression did not significantly predict depression in Model 3, *b* = −0.077, *p* = 0.184, 95% *CI* = [−0.192, 0.037]. We also generated 5000 bootstrapping samples from the original data set (n = 654). But the indirect effect of loneliness on depression via expressive suppression was not significant, *b* = −0.077, *Boot 95% CI* = [−0.205, 0.043], *p* > 0.05. And loneliness was not significantly associated with expressive suppression, *b* = 0.073, *Boot 95% CI* = [−0.008, 0.153]. Therefore, the mediation effect cound not be explained by expressive suppression. Hypothesis 3 was thus supported that only cognitive reappraisal played a mediating role in the relationship between loneliness and depression.

**TABLE 3:** Testing the mediation effect of cognitive reappraisal on depression.

Predictors	Model 1 (depression)			Model 2 (CR)			Model 3 (depression)		
	*b*	*SE*	*t*	*b*	*SE*	*t*	*b*	*SE*	*t*
Gender	–0.459	0.492	–0.934	–0.151	0.410	–0.369	–0.514	0.489	–1.096
Age	–0.082	0.136	–0.603	–0.165	0.114	–1.454	–0.142	0.130	–1.092
Loneliness	0.866***	0.054	16.019	−0.426***	0.045	–9.459	0.711***	0.055	12.930
ERQ: CR							−0.364***	0.045	–8.100
R^2^	0.283			0.124			0.349		
F	85.643***			30.756***			87.018***		

ERQ, Emotion Regulation Questionnaire; CR, cognitive reappraisal. ***p < 0.001.

**TABLE 4:** Testing the mediation effect of expressive suppression on depression.

Predictors	Model 1 (depression)			Model 2 (ES)			Model 3 (depression)		
	*b*	*SE*	*t*	*b*	*SE*	*t*	*b*	*SE*	*t*
Gender	–0.459	0.492	–0.934	–1.678	0.331	–5.066	–0.5894	0.501	–1.176
Age	–0.082	0.136	–0.603	–0.165	0.092	0.168	–0.081	0.136	–0.594
Loneliness	0.866***	0.054	16.019	0.073*	0.036	2.017	0.871***	0.054	16.084
ERQ: ES							–0.077	0.058	–1.329
R^2^	0.283			0.043			0.285		
F	85.643***			9.666***			64.749***		

ERQ: Emotion Regulation Questionnaire; ES, expressive suppression. *p < 0.05. ***p < 0.001.

### Testing for the Moderated Mediation Effect

To examine the relationship between loneliness, emotion regulation (only cognitive reappraisal), and depression, a moderated mediation model was conducted. We conducted moderated mediation analysis using PROCESS windows (Model 59) in SPSS to examine the proposed model (see Figure 1). As shown in Table 5, in Model 1, loneliness was significantly related with cognitive reappraisal, *b* = −0.305, *p* < 0.001. Resilience was significantly related with cognitive reappraisal, *b* = 0.303, *p* < 0.001, but it did not play a moderating role in the relationship between loneliness ans cognitive reappraisal, *b* = −0.011, *p* = 0.253. Model 2 indicated that the relationship of loneliness and cognitive reappraisal with depression, respectively, were moderated by resilience. The conditional indirect effect of loneliness on depression via cognitive reappraisal was significant, *b* = −0.301, *p* < 0.001, *Boot 95% CI* = [−0.388, −0.215]. The conditional direct effect of loneliness on depression was significant, *b* = 0.591, *p* < 0.001, *Boot 95% CI* = [0.485, 0.698]. The conditional indirect effect moderated by resilience was significant, *b* = 0.035, *p* < 0.001, *Boot 95% CI* = [0.014, 0.055]. The conditional direct effect moderated by resilience was also significant, *b* = −0.031, *p* < 0.001, *Boot 95% CI* = [−0.058, −0.005]. Hypothesis 4 was thus supported.

**TABLE 5:** The moderated mediation effect of loneliness on depression.

Predictors	Model 1 (CR)			Model 2 (depression)		
	*b*	*SE*	*t*	*b*	*SE*	*t*
Loneliness	−0.305***	0.049	–6.247	0.591***	0.056	10.547
Resilience	0.303***	0.054	5.604	−0.418***	0.063	–6.644
Loneliness × resilience	–0.011	0.010	–1.144	−0.031**	0.013	–2.383
CR				−0.301***	0.044	–6.805
CR × resilience				0.035***	0.010	3.653
*R* ^2^	0.170			0.414		
*F*	26.554***			65.259***		

CR, cognitive reappraisal. **p < 0.01. ***p < 0.001.

**FIGURE 2:**
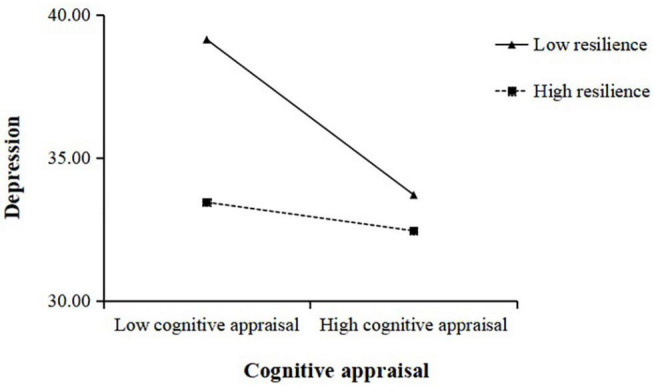
The interaction between cognitive appraisal and resilience and its impact on depression.

**FIGURE 3:**
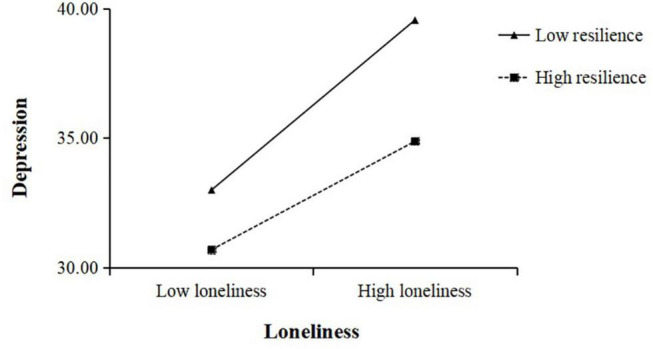
The interaction between loneliness and resilience and its impact on depression.

We plotted depression against cognitive reappraisal separately according to different levels of resilience which was used resilience valued at ± 1SD above and below the mean value (Figure 2). The results of the simple slope tests suggested that lower cognitive reappraisal was negatively correlated with higher levels of depression among low resilience participants, *b* = −0.49, *p* < 0.05. However, the result of cognitive reappraisal and depression was not significant among high resilience participants, *b* = −0.09, *p* > 0.05. Cognitive reappraisal was thus negatively correlated with depression among low resilience participants. However, this association was not significant among high resilience participants. The results of Model 2 also indicated that resilience played a moderating role in the relationship between loneliness and depression. We also plotted depression against loneliness separately according to different levels of resilience (Figure 3). The results indicated that higher levels of loneliness were positively correlated with higher levels of depression among low resilience participants, *b* = 0.72, *p* < 0.001. However, for high resilience individuals, this effect was still significant, though weaker than the low resilience individuals, *b* = 0.46, *p* < 0.01.

The results of bias-corrected percentile bootstrap further indicated that resilience played a moderated role in the relationship between loneliness and depression through cognitive reappraisal. The findings showed that there was a significant indirect relationship between loneliness and depression via cognitive reappraisal among low resilience participants, *b* = 0.19, *SE* = 0.04, 95% *CI* = [0.12, 0.27]. However, this indirect relation was not significant among high resilience participants, *b* = 0.07, *SE* = 0.02, 95% *CI* = [0.02, 0.12]. These results suggested that resilience moderated the path between loneliness and depression and the path between cognitive reappraisal and depression.

In summary, our findings revealed that cognitive reappraisal played a mediating role in the relationship between loneliness and depression and that resilience moderated the association between loneliness and depression while also moderating the impact of cognitive reappraisal on depression.

## Discussion

In this study, the results indicated that loneliness positively correlated with depression in young adults and that this relationship was mediated by cognitive reappraisal. Additionally, the moderation effect revealed that resilience buffered (moderated) these relationships during the COVID-19 pandemic. However, inconsistent with our hypotheses on the moderation effect, we only found that resilience moderated the association between loneliness and depression, and moderated the impact of cognitive reappraisal on depression. Overall, our findings broaden our understanding of loneliness and depression (Cindy et al., 2020; Palgi et al., 2020; Rossi et al., 2020; Groarke et al., 2021) and contribute toward research that links depression with resilience (Zhao et al., 2018; Ye et al., 2020; Salah et al., 2021).

Young adults face a high risk of increased loneliness associated with depression. Our findings were consistent with previous research (Cindy et al., 2020; Palgi et al., 2020; Rossi et al., 2020; Groarke et al., 2021) and indicated that young adults have shown high rates of loneliness during the COVID-19 pandemic (Cindy et al., 2020; Lee et al., 2020; Rhew et al., 2020; Smith and Lim, 2020; Ramo and Lim, 2021). Young adulthood is an important period of cognitive and personality development, and individuals going through this period are vulnerable to mental health problems (Lee et al., 2020; Ramo and Lim, 2021). Loneliness may be one of the worst experiences that young adults encounter, and evidence from different countries indicates that young adults have had the highest increase in rates of psychological distress during the pandemic (Losada-Baltar et al., 2020; McGinty et al., 2020; Pierce et al., 2020; Rossell et al., 2021).

Modeling predictors of depression have identified cognitive reappraisal and resilience as protective factors among young adults. This finding highlights the importance of monitoring cognitive reappraisal and resilience in young individuals. In our study, cognitive reappraisal was found to play a mediating role between loneliness and depression, which indicates that adaptive emotion regulation strategies could be used to reduce perceived loneliness and, subsequently, depression in the context of COVID-19. These findings align with previous studies on trauma (Nickerson et al., 2015; McRae, 2016). Our study showed that cognitive reappraisal is an effective emotional regulation strategy that could change people’s views toward negative events and thus confirmed the cognitive reappraisal theory (McRae, 2016). Therefore, cognitive reappraisal has a mediating impact on emotion in that it allows individuals to assume a positive rather than a negative perspective toward a certain event, which could help them alter their emotional response and promote their mental health (McRae, 2016). In addition, our results also revealed that none of loneliness, depression or resilience in this study was associated with expressive suppression which was consistent with one recent study (Tyra et al., 2021), but inconsistent with prior studies that expressive suppression was significantly associated with higher loneliness (Gubler et al., 2020) and greater depression (Zhang et al., 2021). One possibility was that there was no insufficient time to develop severe depressive symptoms which made the relationship of expressive suppression with loneliness and depression be difficult to be detected at the assessment. Another possibility was that emotion regulation strategies may have different outcomes due to the different situation. At the early stages of the COVID-19 pandemic with very high uncertainty, the assessment may affect by the frequently changing guidelines and restrictions (Wang C. et al., 2020) which could explain why no significant associationwas found between expressive suppression and loneliness or depression.

Our results indicated that resilience is negatively associated with loneliness and depression, which is consistent with previous research findings (Zhao et al., 2018). Thus, resilience and loneliness affect young adults’ risk for depression as a result of the COVID-19 pandemic. Young adults with higher levels of resilience experienced lower levels of loneliness and depression because they were able to cope more successfully when faced with the stressors of the COVID-19 pandemic (Kaye-Kauderer et al., 2021). Studies have found that resilience can help individuals manage negative events (Olsson et al., 2003) and remain optimistic (Guo et al., 2018). As hypothesized, resilience moderated the association between loneliness and depression, which is a finding that aligns with previous studies that have focused on adolescents (Fleming and Ledogar, 2008), and improved the participants’ responses to negative events (Olsson et al., 2003). As we hypothesized based on the resilience theory (Wang et al., 2015), compensatory factors helped neutralize possible negative effects on mental health, even during the COVID-19 pandemic (Yang et al., 2020). Our study also supported previous research that claims resilience plays an adaptive and compensatory role during times of psychological adversity (Olsson et al., 2003; Wang et al., 2015; Reyes et al., 2019). Therefore, Resilience protects against depression caused by loneliness during the COVID-19 pandemic.

In this study, we constructed and tested a hypothetical model based on the findings of previous studies and theories in order to determine if loneliness can be a risk factor of depression. We also examined the mediating role of cognitive reappraisal and investigated whether resilience moderates the mediation model in the context of the COVID-19 pandemic. Clearly, the model revealed that young adults can maintain their mental health during the COVID-19 pandemic if they are resilient and use cognitive reappraisal strategies. Resilience is associated with individuals who have the ability to manage stress (Olsson et al., 2003); however, psychological problems that emerge as the result of continuous exposure to traumatic events, such as death anxiety and negative emotions, are still inevitable (WHO, 2020). Therefore, cognitive reappraisal is one of the most effective strategies that young adults can use to address the negative emotional responses that stem from psychological adversity (McRae, 2016).

Resilience as one of potential moderators is supported by many studies (Grossman et al., 2021; Liu et al., 2021; Sylvia et al., 2021). Our results indicated that resilience might play a moderated role in the relationship between loneliness and depression during the COVID-19 pandemic. When people in high levels of loneliness, individuals with high resilience were less prone to depression than those with low resilience. Thus, resilience exerted a clear moderating effect by attenuating the relationships of perceived loneliness on depression. Furthermore, there were individual differences in the effects of cognitive reappraisal strategies on resilience. When cognitive reassessment strategies were less employed, individuals with low resilience were more prone to depression than those with high resilience. But, when individuals with high resilience than those with low resilience adopt more cognitive reappraisal strategies to regulate negative emotion, they had lower levels of depression. Therefore, the findings further indicated that resilience regulated the mediating effect of cognitive reappraisal between loneliness and depression.

This study had several limitations for interpretability of the findings. First, the main aim of our research was to explore the protective factors of the relationship between loneliness and depression in the context of the COVID-19 pandemic. Though our study explains how loneliness is associated with depression and examines the mediating role of cognitive reappraisal, its generalizability is limited because we only focused on Chinese young adults and the impact of COVID-19 on young adults’ mental health may vary between different countries and cultures. Second, the results ignored the effects of the participants’ distance from COVID-19 outbreak sites, which may have significantly affected the state of their mental health. Third, as a cross sectional study which only offered correlational effect, it could not provide causal relationship among loneliness and depression. It would be necessary to conduct longitudinal study to further examine the causal relationship between loneliness and depression during the COVID-19 pandemic and other trauma event which may develop and lead to mental disorders. Fourth, the effects of these variables before and after the pandemic were not evaluated. This study was conducted during the COVID-19 pandemic, therefore we did not measure the loneliness and depression of the participants before the pandemic. Finally, it was not clear whether the model (see Figure 1) was purely driven by loneliness or it’s a shared mechanism with loneliness and social anxiety (e.g., hypervigilance to social threats). Previous studies have revealed a high correlation between loneliness and social anxiety (Lim et al., 2016, 2022; Eres et al., 2021), but we did not examine this relationship in the current study.

Therefore, further investigation should not only use more representative samples and verify the findings, but also should focus more on the different mechanisms of loneliness and social anxiety on affecting others and explicitly measure the effect that COVID-19 pandemic might bring on the variables (e.g., perceived stress directly triggered by the epidemic). Thus, more psychological constructs, such as social anxiety, social support and self-esteem, may be involved in the future studies to better understand how individuals cope with the adverse consequences of COVID-19 and may respond more adaptively in future pandemics.

The current study revealed that the protective factors of cognitive reappraisal and resilience mediate the relationship between loneliness and depression, which was a finding that could have useful clinical implications. Our research indicated that young adults who use cognitive reappraisal more frequently were able to partially mediate the relationship between loneliness and depression. Moreover, we found that the resilience of young adults not only moderates the relationship between loneliness and depression, but also moderates the effect of cognitive reappraisal on depression. Therefore, the protective factors of cognitive reappraisal and resilience have alleviated the impact of loneliness on depression among Chinese young adults during the COVID-19 pandemic. These findings suggested that governments and educational institutions should cooperate with one another in order to confront the issue of deteriorating mental health among young people and provide timely and effective services that could promote adaptability and positive psychological health among this age group. In summary, our findings shed light on the relationship between loneliness and depression and broaden our understanding of how to use protective factors (such as cognitive reappraisal and resilience) to create public health interventions during the COVID-19 pandemic, especially among young adults.

## Data Availability Statement

The raw data supporting the conclusions of this article will be made available by the authors, without undue reservation.

## Ethics Statement

The studies involving human participants were reviewed and approved by the Institutional Review Board at San Yat-sen University. The patients/participants provided their written informed consent to participate in this study.

## Author Contributions

FL designed the research, analyzed the data, and drafted the manuscript. MY designed the research and drafted the manuscript. JL collected the data and drafted the manuscript. JT, YZ, and YL revised the manuscript. ZY and MX collected the data. SG designed the research. DG designed the research and wrote the manuscript. All authors listed met authorship criteria, certified that they have participated sufficiently in the work to take public responsibility for the content, and approved the final version of the manuscript for submission.

## Conflict of Interest

The authors declare that the research was conducted in the absence of any commercial or financial relationships that could be construed as a potential conflict of interest.

## Publisher’s Note

All claims expressed in this article are solely those of the authors and do not necessarily represent those of their affiliated organizations, or those of the publisher, the editors and the reviewers. Any product that may be evaluated in this article, or claim that may be made by its manufacturer, is not guaranteed or endorsed by the publisher.
